# The Heritage School of Arts and Crafts for Crippled Boys, Chailey, Sussex

**Published:** 1910-10-15

**Authors:** 


### EDITOR'S LETTER-BOX.

### THE HERITAGE SCHOOL OF ARTS AND CRAFTS FOR CRIPPLED BOYS, CHAILEY, SUSSEX.

Sir,—The schools for both boys and girls at Chailey have attracted great attention, both at home and abroad, as a pioneer movement in the education of our cripples. Their object is, by placing these otherwise unhappy children amid bright and healthy surroundings, to inspire them with courage to fight against their handicap in the battle of life, and to equip them to support themselves by a thorough training in suitable crafts. Under the administration of Mrs. Kimmins, the founder of the movement, whose remarkable and energetic devotion has hitherto triumphed over every obstacle, these schools have achieved great success, and have received full recognition by the Board of Education, whose inspectors have given them most gratifying reports. Many of those who have passed through the schools have become independent, and most are earning good wages. The schools are full to overflowing, with long waiting lists.

The girls' school is a new building, the gift of Lord Llangattock, the honorary treasurer. The boys' school has been carried on in an old house, which was only passed by the Board of Education as satisfactory during the experimental stage, but now that the institution has become fully established, it is absolutely necessary to make the buildings suitable for modern requirements. The supporters are, therefore, faced with the necessity of reconstruction, unless they abandon the work begun with so much promise. It is estimated that the necessary alterations and extensions will cost £5,000. Towards this sum a few friends have promised or guaranteed £2,500 conditionally on a sufficient sum being raised; and I feel sure that the crisis has only to be made known to bring forward other helpers.

The President of these schools is the Bishop of London, and the Hon. Treasurers Lord Llangattock and Miss Alice Rennie, 36 Westbourne Terrace, Hyde Park, W., who will be happy to receive contributions.

Yours faithfully,

October 1910. (Signed) Louise.

